# Effect of Si and B on the Electrochemical Behavior of FeCoNiCr-Based High-Entropy Amorphous Alloys

**DOI:** 10.3390/ma15248897

**Published:** 2022-12-13

**Authors:** S. Leila Panahi, Pere Bruna, Eloi Pineda

**Affiliations:** Department of Physics, Institute of Energy Technologies, Universitat Politècnica de Catalunya, 08019 Barcelona, Spain

**Keywords:** corrosion, high-entropy alloys, metallic glasses

## Abstract

The ability to produce high-entropy alloys with an amorphous structure, so-called high-entropy metallic glasses (HEMGs), offers the possibility to produce new compositions with good mechanical properties and resistance to corrosion. In this study, corrosion behavior was studied in two HEMGs, FeCoNiCrB and FeCoNiCr(BSi). In both cases, the total amount of metalloid atoms was kept constant at 20 at.%. The electrochemical behavior of these alloys was studied by means of linear polarization resistance (LPR) measurements and electrochemical impedance spectroscopy in a 3 wt.% NaCl solution. The effect of corrosion was characterized by using X-ray photoelectron spectroscopy (XPS) and the surface morphology was checked using a scanning electron microscope (SEM). The results show that samples with B but without Si exhibit better corrosion resistance due to its chemical homogeneity and lack of structural heterogeneity.

## 1. Introduction

A large variety of amorphous alloys with excellent corrosion resistance have been reported in the literature, e.g., Zr- [1], Fe- [2], Ti- [3], and Ni-based [4] systems. It is well known that chemical composition plays an important role in the corrosion behavior of metallic glasses (MGs). In these kinds of alloys, their high corrosion resistance can be attributed to their compositional and structural homogeneity [5]. Recently, there has been growing interest in producing compositions of metallic glasses with the characteristics of high-entropy alloys (HEAs), the so-called high-entropy metallic glasses (HEMGs) that combine the chemical homogeneity of MGs and HEAs with the disordered structure of MGs. A major amount of the research in the field of HEAs has been focused on alloys consisting of Ni, Co, Al, Cu, Fe, Mn, and Cr [6]. Generally, HEAs exhibit good resistance to wear and corrosion and have a high hardness. Recently, it has been shown that the addition of varying amounts of Cr, Al, Si, and Ti in HEAs improves their mechanical and oxidation resistance by the formation of protective layers of Al_2_O_3_, Cr_2_O_3_, and SiO_2_ [7]. In general, there is a large amount of corrosion studies on crystalline HEAs; see for example a recent review by Nascimento et al. [8], and in some of the studies, it has been shown that the addition of large amounts of B and/or Si can be detrimental to corrosion resistance [9,10]. However, in the field of metallic glasses, the limited addition of metalloids such as P, B, or C is usually beneficial for corrosion resistance [11,12]. In the case of HEMGs, Gong et al. [13] have shown that the improvement in the corrosion resistance can be ascribed to two effects: on the one hand to the reduction in atomic mobility due to the high-entropy effect and, on the other hand, to the cocktail effect where the mixture of several corrosion resistant elements can affect the overall behavior. Following this strategy, we modified the composition of one particular HEA [14,15], the equimolar FeCoCrNi, which consists of a stable face-centered cubic (FCC) solid solution with good plasticity and toughness but with low hardness and poor wear resistance. In order to improve the mechanical and electrochemical resistance of this alloy, B and Si were added to this base composition. For a particular combination of B and Si, the resultant alloys, (FeCoCrNi)_100-x-y_B_x_Si_y_ with x = 0, 5, 10, 15, 20 and y = 0, 5, 10, 15, 20 at.%, can be quenched as HEMGs with a completely disordered structure.

We studied the enhancement of the mechanical properties of these new materials in [14] whereas the behavior of these HEMGs in a corrosive environment is presented here. As far as we know, there are no other works on the system FeCoCrNi besides the one by Ding et al. [16] and there are only corrosion studies on similar systems based on the FeNiCrMo and FeCoNiCrMo HEAs [11,12]. Specifically, in this work, in order to complete the characterization of these FeCoCrNiBSi alloys and to be able to find the best combination of B and Si that globally enhances their mechanical and electrochemical properties, we focus our attention on two particular compositions representative of all the series of alloys: (a) the case with x = 20 and y = 0 that we will label AB20 and (b) the case with x = y = 10 that we will label AB10Si10. The alloy without B or Si (x = y = 0), which is labelled as A, has also been produced as reference. Potentiodynamic polarization tests and electrochemical impedance spectroscopy were used to characterize the passivation behavior of the alloys while scanning electron microscopy (SEM) and X-ray photoelectron spectroscopy (XPS) were performed in order to study their surface.

## 2. Experimental

### 2.1. Materials and Methods

The (FeCoCrNi)_100_, (FeCoCrNi)_80_B_20_, and (FeCoCrNi)_80_B_10_Si_10_ master alloys, which from now on are called A, AB20, and AB10Si10, respectively, were produced by arc-melting of pure elements: iron sheet (99.9 wt.%), cobalt lumps (99.5 wt.%), chromium lumps (99.95 wt.%), nickel wire (99.98 wt.%), boron lumps (99 wt.%), and silicon lumps (99.999 wt.%). In all the compositions, the master alloy was equiatomic in Fe, Co, Cr, and Ni. The arc-melting process was performed on a water-cooled copper plate in a Ti-gettered argon atmosphere to avoid oxidation. The master alloys were remelted three times to assure compositional homogeneity. Afterwards, amorphous ribbons were prepared from the alloys by melt spinning at a linear tangential speed of 40 m s^−1^; the surface of the ribbons in contact with the copper wheel during the quenching process will be termed here as the ‘wheel side’ while the other surface will be termed as the ‘free side’. The thickness and the width of the resulting ribbons were of 20–30 μm and 4 mm, respectively. Prior to the electrochemical measurements, all the samples were ultrasonically cleaned with acetone and afterwards with distilled water before drying. Corrosion tests for the as-prepared samples were performed on the free side of the ribbons which acted as a working electrode. For these measurements the ribbons were connected to a pure copper (99.9 at.%) wire to obtain an electrical connection and the copper wire and the wheel side of the ribbons were covered by PTFE tape.

### 2.2. Potentiodynamic Measurements

Electrochemical tests were performed in a three-electrode corrosion cell in a 3 wt.% (0.51 M) NaCl solution at 25 °C. The reference electrode was Ag/AgCl (3.5 M in KCl), thus 0.205 V should be added to convert the potential values to the scale where a saturated hydrogen electrode at 25 °C has a potential of zero, and a spiral platinum wire was used as a counter electrode. The surface of the specimens exposed to the solution was around 0.08–1.31 cm^2^. The measurements were performed with a SP-200 (Bio-Logic Science Instruments, Seyssinet-Pariset, France) potentiostat/galvanostat operated by the EC-Lab software. In the first step, the specimens were stabilized for 1 h to obtain a relatively stable open circuit potential (OCP) followed by potentiodynamic polarization curves between the OCP value minus 0.1 mV and the OCP value plus 0.8 V at a potential scan rate of 0.1 mV/s. All measurements were performed at least three times to assure replicability.

### 2.3. Electrochemical Impedance Spectroscopy

Electrochemical impedance spectroscopy (EIS) measurements were performed at open circuit conditions in the frequency range from 100 kHz to 100 mHz with the SP-200 potentiometer (Bio-Logic Science Instrument, Seyssinet-Pariset, France). The AC excitation voltage was 10 mV (rms). The EIS spectra were recorded after 1 h at the OCP.

### 2.4. Surface Characterization

The samples were analyzed using a scanning electron microscope (SEM) at an electron beam energy of 15 keV at a Neon40 Crossbeam™ workstation (Carl Zeiss, Hamburg, Germany). The X-ray photoelectron spectroscopy (XPS) was performed with a SPECS system equipped with a Phoibos 150 MCD-9 detector and an Al anode XR50 source working at 150 W. For the high-resolution spectra, a scan step of 0.1 eV was applied. The sample spot analyzed had a diameter of 1 mm^2^ and the Casa XPS program (Casa software Ltd., Teignmouth, United Kingdom) was used to evaluate the data. The spectra were normalized on the binding energy scale relative to the position of C 1 s peak at 284.8 eV and the intensity of the adventitious carbon signal was not taken into consideration. The shown spectra are not normalized on the intensity scale.

## 3. Results and Discussion

### 3.1. Electrochemical Measurements in NaCl Solution

**Figure 1:**
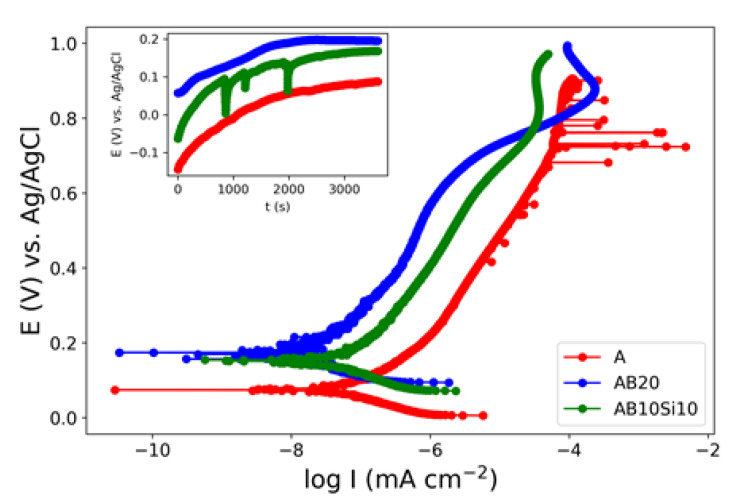
Potentiodynamic polarization curves of as-quenched A, AB20, and AB10Si10 ribbons in 3 wt.% NaCl. The inset show the evolution with time of the OCP.

The potentiodynamic polarization curves for the A, AB20, and AB10Si10 samples in 3 wt.% NaCl solution are shown in Figure 1. The corrosion potential (E_corr_) and corrosion current density (I_corr_) were determined by extrapolating the Tafel curves and are summarized in Table 1. The values of E_corr_ and I_corr_ of the as-quenched ribbons change with the amount of B and Si. Here, AB20 has the highest E_corr_ and lowest I_corr_. With the reduction in B and increase in Si, the E_corr_ decreases while I_corr_ is kept constant. The crystalline ribbon without B and Si, which we take as the reference material, has the lowest E_corr_. It is well known that corrosion is more likely to occur at grain boundaries, defects, and regions where there is large segregation of elements, thus the amorphous nature of the AB20 and AB10Si10 samples promotes chemical and microstructural homogeneity and improves the corrosion resistance [17]. Moreover, the action of B as a corrosion inhibitor has been reported by several authors [2,18]. Accordingly, the E_corr_ value of the AB10Si10 is slightly lower than the one of the AB20 alloy, with the same corrosion current density. These electrochemical parameters can be compared with the ones corresponding to other high-entropy alloys. If we compare them with crystalline HEA, the results shown here present a more noble E_corr_ and lower I_corr_ [19,20,21,22] and they also show an improvement with respect to other similar high-entropy bulk metallic glasses that show E_corr_ values between −23 and 77 mV [11].

At this point, a question arise about which factor plays a more important role in corrosion resistance. As commented on previously, some studies explain the improvement in the corrosion resistance because of the high-entropy effect that reduces the mobility of the atoms [13]. We can compute the entropies of these alloys and distinguish between the configurational entropy and the mismatch entropy [23]; the configurational entropy is simply proportional to the ln(N), where N is the number of components, thus we have an increase in this entropy from the A alloy (N = 4) to the AB10Si10 alloy (N = 6). The mismatch entropy has been shown to be proportional to the delta parameter that is a measure of the atomic size difference between the constituent atoms, and this value increase from 0.3% for the A alloy to 14.70% for the AB20 and 10.72% for AB10Si10 [15]. Therefore, from these values and the electrochemical parameters, it would seem that the mismatch entropy may be a factor affecting the corrosion resistance, but this analysis does not consider the fact that AB20 and AB10Si10 are amorphous samples with a disordered structure. To clarify this point a systematic analysis of several families of HEAs and HEMGs should be performed but this is out of the scope of this paper.

### 3.2. Electrochemical Impedance Spectroscopy (EIS) Measurements

**Figure 2:**
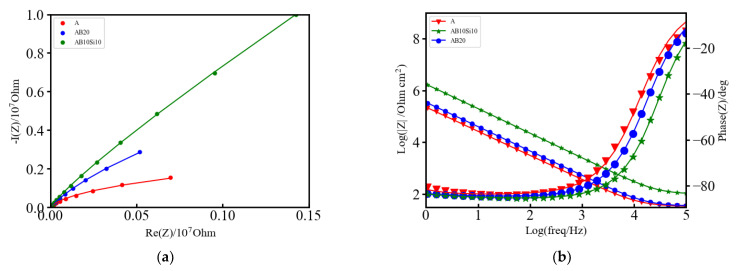
(**a**) Nyquist plot of A, AB20, and AB10Si10 impedance fitted by the equivalent circuit model. Raw data are dots, and the fitting result is represented by lines of the same color and (**b**) Bode plot of A, AB20, and AB10Si10.

**Figure 3:**
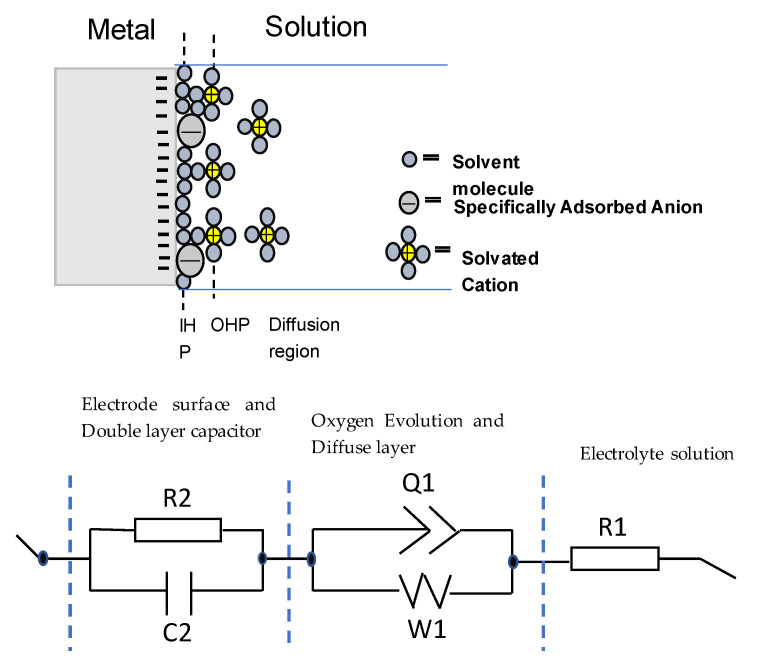
Schematic representation of a metal electrode surface in the solution and the equivalent electrical circuit used to fit the impedance data. R1 and R2 are the electrolyte and charge transfer resistance, respectively. Q1 is the constant phase element, W1 is a Warburg element, and C2 is a capacitor.

Figure 2a,b shows the Nyquist and Bode plots, respectively, of the EIS measurements on the three samples. The absolute value of the impedance (|z|) at very low frequencies (ω → 0) represents the polarization resistance (R_p_), which is the transition resistance between the electrodes and the electrolyte, while the value at very high frequencies (ω → ∞) represents the solution resistance (R_s_) [24]. As can be seen in Figure 2a, the three samples show similar behavior. The phase angle shown in Figure 2b and defined as:

(1) $$\Phi \;=\;\tan^{-1}(\frac{Im\left(Z\right)}{Re\left(Z\right)}),$$

presents some differences. The broadened base of the phase angle and its magnitude are a signal of the pseudo-capacitive behavior, which can be modelled by using a constant phase element [24,25]. Here the single local minimum indicates the presence of a capacitance element that can be modelled by a single capacitor or two capacitors of similar magnitude. For all of these three alloys, these capacitors can be passive films or an electrical double layer capacitance. In order to quantitatively assess the differences between the alloys, the Nyquist and Bode plots have been fitted to an equivalent circuit consisting of one resistor in series with a parallel combination of a constant phase element and a Warburg element and also in series with a parallel combination of a resistor and a capacitor as illustrated in Figure 3. The fitting was performed with EC-Lab v11.10 software and the obtained parameters from the fitting are provided in Table 2. The left part of the circuit, which contains the resistor R2 and the capacitor C2, is related to the movement of mobile charges trough the solid and liquid phases and the non-faradic charge accumulation at the solid/liquid interface of the electrode.

This charge accumulation in the interface constitutes what is commonly known as the capacitive double layer (shown in Figure 3) where three different regions can be distinguished: a first layer that mainly contains polar water molecules and adsorbed anions, called the inner Helmholtz plane (IHP), a second layer with fully hydrated cations, called the outer Helmholtz plane (OHP) and a final diffuse layer composed of hydrated anions and cations [26]. From the values of Table 2, it is evident that the amorphous alloys present higher corrosion resistance as the value of the capacitance is the lowest with respect to the crystalline one. Therefore, it can be stated that in the amorphous alloys, the formation of the double layer capacitance increases the corrosion resistance. However, the overall electrochemical behavior also depends on the inhomogeneities of the electrode surfaces.

Electrode surface irregularities play a significant role in the electrochemical response. There are several factors which cause these irregularities, such as surface roughness and chemical heterogeneities (that include differences in the constituent elements, chemical impurities, and surface band impurities and coatings). Moreover, solid electrodes are not smooth; they exhibit complex surface morphologies with a varying degree of irregular interfaces (i.e., rough, porous, and partially active interfaces). All these effects are modelled by the middle part of the equivalent circuit, which contains a constant phase element (Q1) that corresponds to inhomogeneities in the surface on the atomic and nanoscopic scale (roughness) and crystallographic disorder (due to anisotropic surface atomic structure) of the metal oxide electrode, and a Warburg diffusion element (W1) that corresponds to the diffusion of mobile charges within the metal oxide electrode in the solution. The higher values of Q1 for the A alloy reflect the higher inhomogeneity of this alloy and its lower resistance to corrosion. Finally, the right part of the circuit, R1, is related to the resistance of the electrolyte solution that in this case is higher for the AB10Si10 alloy reflecting better electrochemical behavior.

### 3.3. XPS Analysis of A, AB20, and AB10Si10 Alloys Immersed in NaCl

**Figure 4:**
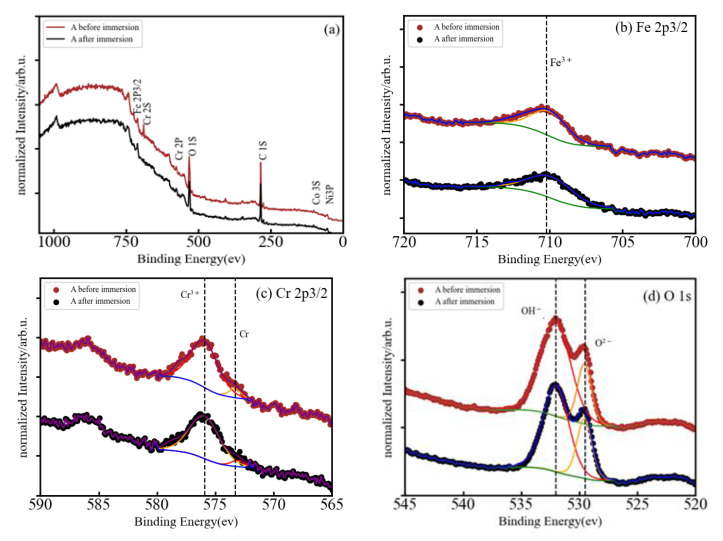
(**a**) Normalized XPS spectrum of A after immersion in 3 wt.% NaCl, and (**b**–**d**) high-resolution XPS spectra of Fe 2p3/2, Cr 2p3/2, and O 1s.

**Figure 5:**
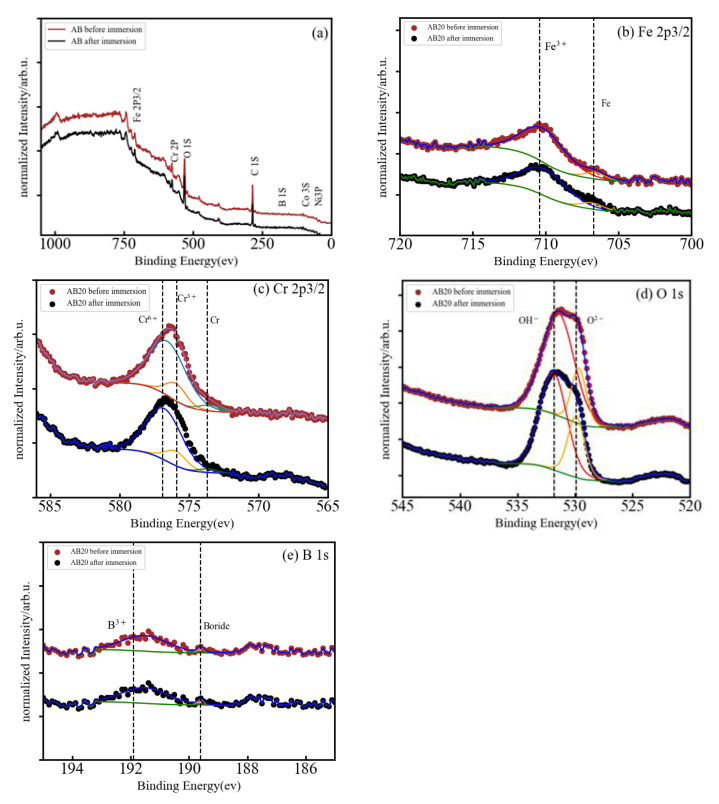
(**a**) Normalized XPS spectrum of AB20 after immersion in 3 wt.% NaCl, and (**b**–**e**) high-resolution XPS spectra of Fe 2p3/2, Cr 2p3/2, O 1s, and B 1s.

**Figure 6:**
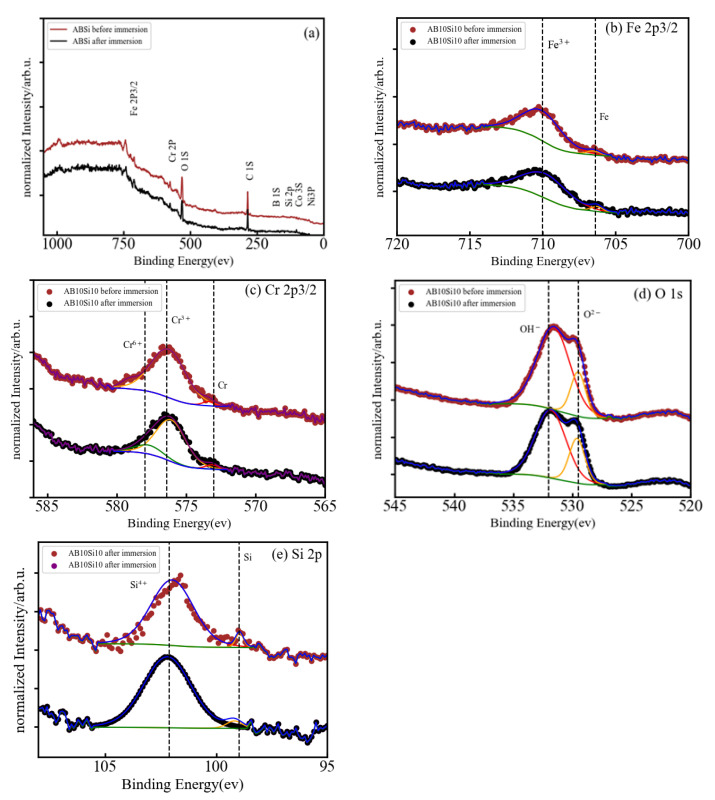
(**a**) Normalized XPS spectrum of AB10Si10 after immersion in 3 wt.% NaCl, and (**b**–**e**) high-resolution XPS spectra of Fe 2p3/2, Cr 2p3/2, O 1s, and Si 2p.

The corrosion resistance of HEMGs in a particular corrosion environment will be determined by how their surface reacts to the corrosion agent and by the composition of the passive film that can be formed on the surface. Therefore, X-ray photoelectron spectroscopy (XPS) is used in this work to obtain information about the effect of anode polarization on the surface composition and determine the oxidation state of the different elements [27]. The use of small amounts of Al, Cr or Si was shown to improve oxidation resistance in conventional alloys due to the formation of a stable oxide layer on the surface [28]. Fe-based HEAs do not present solubility restrictions on the amount of other elements and, thus, Al, Cr or Si may be added in higher concentrations to facilitate the formation of the oxide films [29]. The XPS spectra of our samples are shown in Figure 4a, Figure 5a, and Figure 6a for the A, AB20, and AB10Si10 samples, respectively. The figures show the survey spectrum (low-resolution measurements in a large energy range) in panel (a) and the high-resolution emission peaks associated with the Fe 2p, Cr 2p, and O 1s core electron levels in panels (b), (c), and (d), respectively. Moreover, Figure 5 and Figure 6 also include in panel (e) the B 1s and the Si 2p core electron level emission peaks, respectively. The different dashed lines mark the theoretical energy of different oxidation states of the corresponding element and are used as a reference. The C 1s line is due to the carbon present in the sample holder and is used for the energy calibration. The presence of O in the long-range energy spectra of the three alloys is observed before and after polarization measurements, suggesting that an oxide film was spontaneously formed on the sample surface before immersion in the NaCl solution. Figure 4a shows the normalized XPS spectrum of sample A after immersion in a 3 wt.% solution of NaCl, indicating the presence of Fe, Cr, Co, Ni, and O, although the peaks associated to Co and Ni are barely visible. High-resolution spectra at the energy of the Fe 2p peak reveals that Fe is present on the surface as Fe^3+^ with a binding energy of 710.15 eV (Figure 5b). On the other hand, the Cr 2p peaks are at 573.29 and 575.91 corresponding to metallic Cr and Cr^3+^, respectively (Figure 5c). The presence of Cr^3+^ is considered to be crucial to the quality of the passivation film for the A composition. In addition, the binding energy peaks of O 1s represents a M-O compound characteristic peak corresponding to O^−2^ species (529.54 eV) and a M-(O-H)_n_ compound characteristic peak corresponding to O-H species (532.06 eV) (Figure 4d).

Figure 5a presents the normalized XPS spectrum of the AB20 high-entropy metallic glass alloy before and after immersion in a 3 wt.% NaCl solution. The peaks of Fe 2p, Cr 2p, O 1s, and B 1s can be easily seen and the peaks of Co 3s and Ni 3p are barely visible. The Fe 2p (Figure 5b) presents two peaks centered at 706.81 and 710.09 eV which correspond to metallic Fe and Fe^3+^, respectively. In the Cr 2p XPS spectrum (Figure 5c), the binding energy peaks are centered at 573.53, 575.94, and 576.88 eV and can be ascribed, respectively, to metallic Cr, Cr^3+^, and Cr^6+^. Deconvolution of the O 1s peak (Figure 5d) results again in two component peaks with energies of 529.90 eV that represents a M-O compound characteristic peak corresponding to O^2-^ species and a second peak (531.87 eV) that represents a M-(O-H)_n_ compound characteristic peak corresponding to O-H. B 1s spectra (Figure 5e) was also deconvoluted in two peaks which were assigned to a boride (189.82 eV) and B^3+^ (191.73 eV) bonds, respectively.

In Figure 6a, the full XPS spectra for sample AB10Si10 clearly shows the coexistence of elemental Fe, Cr, O, and Si, while Co, Ni, and B are indistinguishable from the baseline. The high-resolution XPS spectra of Fe 2p, Cr 2, O 1s, and Si 2p are shown in Figure 6b–e. In the Fe 2p XPS spectrum, the binding energy peaks at 706.42 and 710.02 eV are attributed to metallic Fe and Fe^3+^, respectively. There are three peaks of Cr 2p after immersion in solution (Figure 6c) at 573.23 eV that corresponds to metallic Cr, at 576.25 eV for Cr^3+^, and at 577.99 eV for Cr^6+^. Moreover, the high-resolution XPS spectrum of O 1s (Figure 6d) comprises a peak at 529.52 eV for M-O and 531.61 eV for M-(O-H)_n_. The Si 2p spectrum (Figure 6e) shows two peaks centered at 98.96 eV and 101.66 eV that correspond to metallic Si and Si^4+^, respectively.

In summary, in each of the samples almost all the elements were detected on the surface, both in form of oxides and unoxidized metal (M^0^) species. As the XPS signal can gather information from 5–10 nm in depth [30], the significant absence of Co and Ni in the survey spectra means that these two elements do not play any role in the formation of the oxide layer before immersion. The metal oxides Fe^3+^ and Cr^3+^ were detected on the surface of all samples, and it is well known that these oxides can produce a certain level of protection in corrosive environments [31,32]. These two oxides are the dominant oxide forms on the surface of the A, AB20, and AB10Si10. Nascimento et al. [23] explained the high corrosion resistance of the FeCoCrNi alloy by the high concentration of Cr_2_O_3_ in the passive film. The addition of B to the A alloy results in the formation of B-O bonds on the surface of the AB20 sample, while the decrease in B and the addition of Si yields the presence of silicates and a small fraction of metallic Si on the surface of the AB10Si10 alloy. Moreover, and without needing to perform a quantitative analysis, it is clear that for each sample, the oxidized form of each constituent element has a higher percentage than the corresponding unoxidized metal (M^0^) species. According to Qiu et al. [31], it is expected that the coexistence in the complex surface film of both metallic oxides and unoxidized metal species can increase the passivity of the samples and, thus, increase the corrosion resistance. Moreover, some research shows that boron addition not only improves the glass-forming ability of Fe-Cr-B-based alloys but also promotes superior corrosion resistance [33], which is in accordance with the results presented here. On the other hand, replacing the B with Si in AB20, which has the highest E_corr_, favors the creation of silicates on the surface of AB10Si10, which according to Masumoto et al. [9] decreases the corrosion resistance of the alloy, as has been observed in the above LPR results in a 3 wt.% NaCl solution.

### 3.4. Microstructure Characterization after Immersion in NaCl

**Figure 7:**
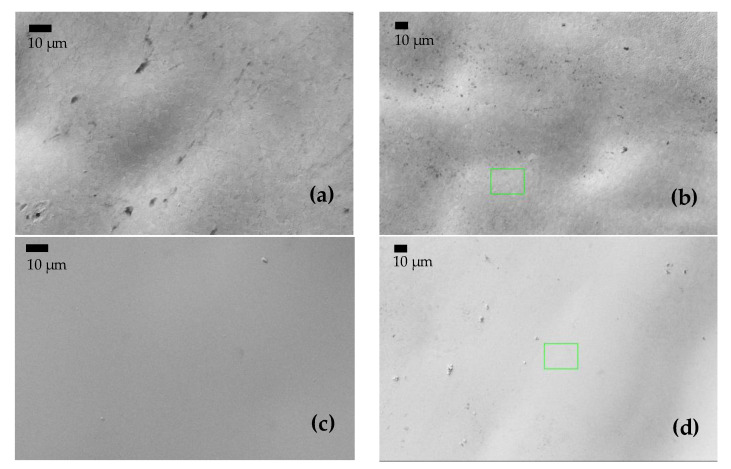

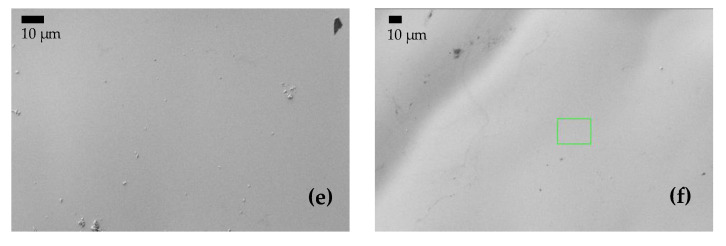
(**a**,**c**,**e**) BSE images of the surface of samples A, AB20, and AB10Si10, respectively, before immersion in 3 wt.% NaCl at room temperature. (**b**,**d**,**f**) BSE images of A, AB20, and AB10Si10, respectively, after immersion.

To gain a better understanding of the evolution of the corrosion product layer after the immersion tests, SEM images were taken to analyze the surface morphology of the alloys. After the polarization experiments, the specimens were cleaned with distilled water and dried in nitrogen immediately. The morphology of samples A, AB20, and AB10Si10 before and after immersion in a 3 wt.% NaCl solution and the linear polarization measurements are shown in Figure 7. Before immersion, the amorphous samples are completely homogenous. In Figure 7b, which corresponds to the A alloy after immersion, we can see some localized corrosion in different points (small black points) which have been damaged by the chloride ion attack, presumably in an area with a low Cr content [34]. Moreover, it should be mentioned that within the sensitivity of the method no inhomogeneities in the element distribution were seen in the FeCoCrNi alloy. Therefore, the passive films that are formed are relatively weakly bonded and inadequate to restrict the propagation of the corrosion process in this system [35,36]. As can be seen from Figure 7d,f, no pitting corrosion exists on the surface of the amorphous samples, which is mainly due to the homogeneous formation of the passive film produced on the surface by the borides and the possible mixture of oxides and hydroxides phases, as seen by XPS. Moreover, the formation of this homogenous passive film is favored by the homogenous amorphous structure. In order to better understand this passive film, a TEM study should be performed.

## 4. Conclusions

The electrochemical properties and the effect of the content of B and Si in (FeCoCrNi)_80_B_10_Si_10_, (FeCoCrNi)_80_B_20_, and FeCoCrNi alloys were studied by linear polarization resistance and electrochemical impedance spectroscopy techniques. The results show that amorphous samples exhibit higher corrosion resistance due to the fact that B promotes the stability of the passivation films. The higher corrosion resistance of (FeCoCrNi)_80_B_20_ may be attributed to the absence of crystallographic defects and the uniformity of the passive film, as well as to the intrinsic higher activity of the amorphous surface. With the reduction in B and the increase in Si, the E_corr_ slightly decreases while I_corr_ is kept constant. The crystalline ribbon without B and Si, that we take as a reference, has a lower E_corr_ than the amorphous ribbons and this can be a consequence of the crystalline FCC structure. From the XPS results it can be concluded that the formation of different combinations of oxides and hydroxides protect the samples from corrosion.

## Figures and Tables

**Table 1:** Summary of the quantitative analysis of the potentiodynamic polarization test of the A, AB20, and AB10Si10 samples. The estimated error for the corrosion potential is ±10 mV while for the corrosion current is ±0.01 nA cm^−2^.

Alloy	E_corr_ (mV)	I_corr_ (nA cm^−2^)
A	70	0.09
AB20	170	0.06
AB10Si10	150	0.06

**Table 2:** Parameters from the fitting of the Nyquist plot to an equivalent electrical circuit.

Alloy	R1 (Ω)	R2 (KΩ)	C2 (F)	Q1 (F s^(a−1)^)	a	W1 (Ω s^−1/2^)
A	33.45	1149	1.697 × 10^−6^	1.335 × 10^−6^	0.906	3.219 × 10^6^
AB20	34.41	21,310	0.854 × 10^−6^	0.991 × 10^−6^	0.913	1.844 × 10^6^
AB10Si10	100.4	258,800	0.156 × 10^−6^	0.200 × 10^−6^	0.927	5.543 × 10^6^

## Data Availability

The raw/processed data required to reproduce these findings can be shared upon request to the corresponding author.
